# The *AAP* gene family for amino acid permeases contributes to development of the cyst nematode *Heterodera schachtii* in roots of Arabidopsis^☆^

**DOI:** 10.1016/j.plaphy.2013.05.016

**Published:** 2013-09

**Authors:** Abdelnaser Elashry, Sakiko Okumoto, Shahid Siddique, Wolfgang Koch, David P. Kreil, Holger Bohlmann

**Affiliations:** a Division of Plant Protection, Department of Crop Sciences, University of Natural Resources and Life Sciences, Vienna. UFT Tulln, Konrad Lorenz Str. 24, 3430 Tulln, Austria; b Department of Plant Pathology, Physiology, and Weed Science, 549 Latham Hall (0390), Virginia Tech, Blacksburg, VA 24061, USA; c KWS SAAT AG, Grimsehlstrasse 31, 37574 Einbeck, Germany; d Chair of Bioinformatics, Department of Biotechnology, University of Natural Resources and Life Sciences, Vienna, Austria; e School of Life Sciences, University of Warwick, UK

**Keywords:** Amino acid transporter, Syncytium, *Heterodera schachtii*, Arabidopsis, Roots

## Abstract

The beet cyst nematode *Heterodera schachtii* is able to infect Arabidopsis plants and induce feeding sites in the root. These syncytia are the only source of nutrients for the nematodes throughout their life and are a nutrient sink for the host plant. We have studied here the role of amino acid transporters for nematode development. Arabidopsis contains a large number of different amino acid transporters in several gene families but those of the *AAP* family were found to be especially expressed in syncytia. Arabidopsis contains 8 *AAP* genes and they were all strongly expressed in syncytia with the exception of *AAP5* and *AAP7*, which were slightly downregulated. We used promoter::GUS lines and *in situ* RT-PCR to confirm the expression of several *AAP* genes and *LHT1*, a lysine- and histidine-specific amino acid transporter, in syncytia. The strong expression of *AAP* genes in syncytia indicated that these transporters are important for the transport of amino acids into syncytia and we used T-DNA mutants for several *AAP* genes to test for their influence on nematode development. We found that mutants of *AAP1*, *AAP2*, and *AAP8* significantly reduced the number of female nematodes developing on these plants. Our study showed that amino acid transport into syncytia is important for the development of the nematodes.

## Highlights

• Arabidopsis *AAP* genes for amino acid permeases are strongly expressed in syncytia.
• Mutants of *AAP1*, *AAP2*, and *AAP8* reduced the number of developing female nematodes.
• Amino acid transport into syncytia is important for the development of the nematodes.

## 1 Introduction

Nematodes are a large group of animals with different life styles, including free-living bacterial feeders such as the model nematode *Caenorhabditis elegans* as well as a variety of pathogens of plants and animals. Obligate biotrophic plant parasitic nematodes attack mainly the roots of many plant species and cause severe damage to their host plants, either directly or as vectors of plant viruses. It has been estimated that the worldwide crop losses due to nematode damage amount to more than $100 billion per year [1]. Some of the most economically important species belong to the family Heteroderidae and induce the formation of specialised feeding sites which are their sole source of nutrients throughout their life. Root-knot nematodes of the genus *Meloidogyne* induce a feeding structure which is composed of several giant cells [2] while cyst nematodes (genera Heterodera and Globodera) induce a feeding structure which is a syncytium. Cyst nematodes enter the plant roots as second stage juveniles (J2) and select a single root cell to induce a syncytium, which then expands by incorporation of up to several hundred neighbouring cells by local cell wall dissolution. The adult male cyst nematodes leave the roots to mate with females. After fertilization, the female cyst nematode continues to feed from the syncytium until the egg development is completed. The dead body, which is then called a cyst, protects several hundred eggs until infective J2 hatch in favourable conditions. Cysts can survive in the soil for many years which makes the cyst nematodes difficult to control in agriculture. The sugar beet cyst nematode *Heterodera schachtii* completes its life cycle on Arabidopsis roots *in vitro* within six weeks [3] and this interaction has been established as a model system. Arabidopsis can be cultured on artificial media under sterile conditions in Petri dishes and the translucent roots facilitate the study of the development of the nematodes inside the root.

The drastic changes in cell morphology of syncytial elements [4,5] as compared with normal root cells imply an underlying global change in gene expression and a variety of methods were used to identify genes that are specifically induced in syncytia or in giant cells (reviewed by Gheysen and Fenoll [6]). We have recently analysed the transcriptome of syncytia induced by *H. schachtii* in roots of Arabidopsis at 5 and 15 days post-inoculation (dpi) [7] and found that one of the strongly induced genes in syncytia coded for the amino acid permease AAP8.

Amino acids represent one of the essential long-distance transport forms for the distribution of organic nitrogen in plants. This allocation of amino acids is mediated by both xylem and phloem [8]. In the xylem, the transport occurs unidirectionally upwards, whereas in the phloem the translocation is bidirectional and the nutrients flow from source to sink tissues. On their way from the sites of uptake and biosynthesis in roots or leaves, amino acids have to be transported across several membranes to enter the long-distance pathways or to reach sink tissues. Amino acid translocation thus requires proteins which control the transport across these membranes.

By functional analysis and sequence homology, a large number of potential amino acid transporters from different gene families were found in the Arabidopsis genome [9,10]. The most important groups are amino acid permeases (AAP), cationic amino acid transporters (CAT), and lysine/histidine transporters (LHT), which all mediate proton-dependent import of amino acids into the cell [11–18]. Prior to a proton coupled import, amino acids have to be exported into the apoplast. This is especially required where no intracellular connections, such as plasmodesmata, are present. An apoplastic pathway exists, for example, in roots, where passive diffusion between epidermis and cortex cells ends at the casparian strip of the endodermis [19] and solutes must enter the symplast. Finally, the nutrients need to be exported out of the symplast into the tracheary elements, as these are dead by maturity and therefore belong to the apoplast. So far, only few plant proteins have been published that mediate a bidirectional transport, and, hence, also an efflux of amino acids. *At*BAT1 [20] was shown to mediate the efflux of glutamate and lysine, but also the influx of alanine and arginine. Another Arabidopsis membrane protein, SIAR1 [21] has been shown to play an important role in organic nitrogen allocation and particularly in amino acid homeostasis in developing siliques.

Physiological functions have been proposed for several amino acid transporters. The import of amino acids into the filial part of the seeds is most likely mediated by members of the AAP family. The expression of the high affinity transporter AAP1 was detected in embryos and is responsible for the import of amino acids into the filial tissue [11,13,22–24]. Besides AAP1, other secondary active amino acid importers were identified to be involved in the amino acid uptake into developing seeds such as AAP2 and AAP8 [25,26].

Microsporogenesis represents a major sink for nitrogen [18]. The situation in stamen resembles the one in developing seeds as the filaments contain a strand of vascular tissue which ends at the connective. Thus, the delivery of nutrients to the anthers must occur via an unloading process and a subsequent transfer across apoplastic barriers towards the developing pollen grains. The uptake of neutral and acidic amino acids into tapetum cells is dependent on LHT2 [18,27]. Amino acid uptake is also essential in symbiotic interactions with mycorrhizal fungi and rhizobia [8] and in plant–pathogen interactions. Amino acid transporters are, for instance, specifically expressed in haustoria which are produced by biotrophic fungal pathogens for the uptake of nutrients from plant cells [28].

Amino acids supply is also important in the pathogenic interaction between nematodes and plant roots. Syncytia and also giant cells, feeding sites induced by several genera of sedentary plant pathogenic nematodes, have a high metabolic activity and are a severe sink for the plant since the nutrients that are taken up by the nematode have to be continuously restored. Amino acids and other nutrients must either be taken up from the apoplast with the help of specific transport proteins or provided symplastically through plasmodesmata. It was originally thought that syncytia are symplastically isolated [29] but, recently, evidence has been reported that there is a direct connection between syncytia and the phloem [30]. Still, the apoplastic pathway seems to play an important role for nutrient uptake into syncytia since several genes for sugar transporters are induced in syncytia and are important for nematode development [31]. Besides sugars, syncytia also have to take up amino acids as a nitrogen source to cope with the constant loss due to nematode feeding. Indeed, the level of 14 amino acids was higher in syncytia as compared with uninfected roots and with root tissues surrounding the syncytium [32]. Correspondingly, our recent transcriptome analysis has revealed that several AAP amino acid transporter genes are strongly upregulated in syncytia [7]. A similar situation has been found in giant cells induced by the root-knot nematode *Meloidogyne incognita* in Arabidopsis roots. A microarray analysis of root sections containing root knots showed that the expression of many amino acid transporters was significantly altered as compared to control root sections [33,34].

Here we report our expression analysis of AAP-type amino acid transporter genes in syncytia. In addition, we included the *LHT1* gene which is also expressed in syncytia and roots at a high level. To test the importance of these genes for the development of the nematodes we used the available knock-out mutants.

## 2 Results

We recently performed a transcriptome analysis of syncytia induced by *H. schachtii* in Arabidopsis roots [7]. *AAP8* was one of the genes that was found to be strongly upregulated in syncytia as compared to control root sections and this was confirmed by real-time RT-PCR and *in situ* RT-PCR. Here we have extended that work and have in addition studied the expression of several other AAP-type amino acid transporter genes together with *LHT1*. As shown in Table 1, 6 of the 8 *AAP* genes are expressed at high levels in syncytia and most of them are upregulated as compared to control root sections. Only *AAP5* and *AAP7* of the 8 *AAP* genes are slightly downregulated. Of the other 44 amino acid transporter related genes, only *LHT1* showed a very strong expression in roots and in syncytia and was therefore included in this study. However, it was approximately three folds downregulated as compared to control root sections. Among the other amino acid transporter related genes, only rather few were expressed at a higher level in syncytia than in control root sections (Table 1) which is also evident from Fig. S1. Comparing expression in 5 and 15 dpi syncytia, only *AAP8* showed a significantly different expression, being approximately expressed 10 folds higher in 15 dpi syncytia (Fig S2). These data indicate that the AAP-type gene family might be especially important for the amino acid uptake into syncytia. We have therefore in addition studied their expression using promoter::GUS lines and *in situ* RT-PCR.

### 2.1 Promoter::GUS analysis

**Fig. 1:**
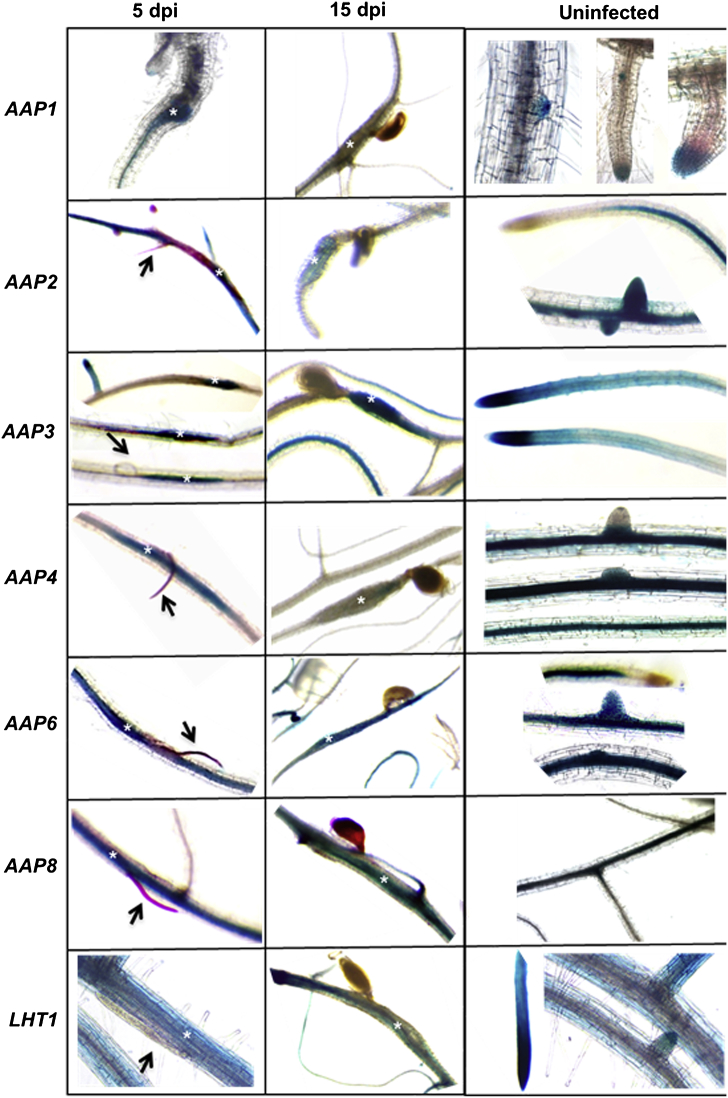
GUS analysis of infected and uninfected Arabidopsis roots of *pAAP*::GUS fusion lines. Uninfected roots and syncytia were stained for GUS activity at 5 and 15 dpi, respectively. The nematodes at 5 dpi were stained with acid fuchsin to ease observation and juveniles are marked with arrows.

The GUS staining results of the promoter::GUS lines for *AAP1*, *AAP2*, *AAP3*, *AAP4*, *AAP6*, *AAP8*, and *LHT1* (Table S1), as related to nematode infection, are shown in Fig. 1. *AAP1* showed GUS staining that was limited to lateral root tips and emerging lateral root primordia. There was no GUS staining observed in syncytia at both 5 and 15 dpi. *AAP2* showed no expression at the root tip and the elongation zone. *AAP2* expression appeared in the maturation zone and further posterior and it showed a higher expression level in 5 dpi syncytia in comparison with the surrounding tissues and it showed a faint staining in 15 dpi syncytia. The expression of *AAP3* was strongest in root tips, root meristem, and elongation zone and the expression decreased in older parts of the lateral roots. *AAP3* showed a strong GUS expression in 5 dpi and 15 dpi syncytia with no staining of the surrounding root part. *AAP4* showed a similar expression pattern as *AAP3* in 5 dpi and 15 dpi syncytia. However, *AAP4*::*GUS* syncytia at 15 dpi showed a fainter staining than in *AAP3* 15 dpi syncytia. *AAP6* and *AAP8* did not show expression in the early primordia and the elongation zone but in the older parts of the roots. Both *AAP6* and *AAP8* showed a strong expression in syncytia at 5 dpi and 15 dpi. *LHT1* gave a strong GUS staining in all root parts, especially in the root tips, while GUS staining in syncytia at 5 dpi and 15 dpi was less than in the surrounding root tissues.

All the GUS lines except *AAP4*::*GUS* have been studied before (for references see Table S1) but none of these studies looked at the expression in syncytia. We have therefore restricted our work to the above-mentioned expression studies of syncytia and control roots. However, *AAP4*::*GUS* has not been reported before and we have included GUS pictures from leaves in the supplement (Fig. S3). It is evident that the expression is restricted to the leave veins.

### 2.2 Localization of gene expression by *in situ* RT-PCR

**Fig. 2:**
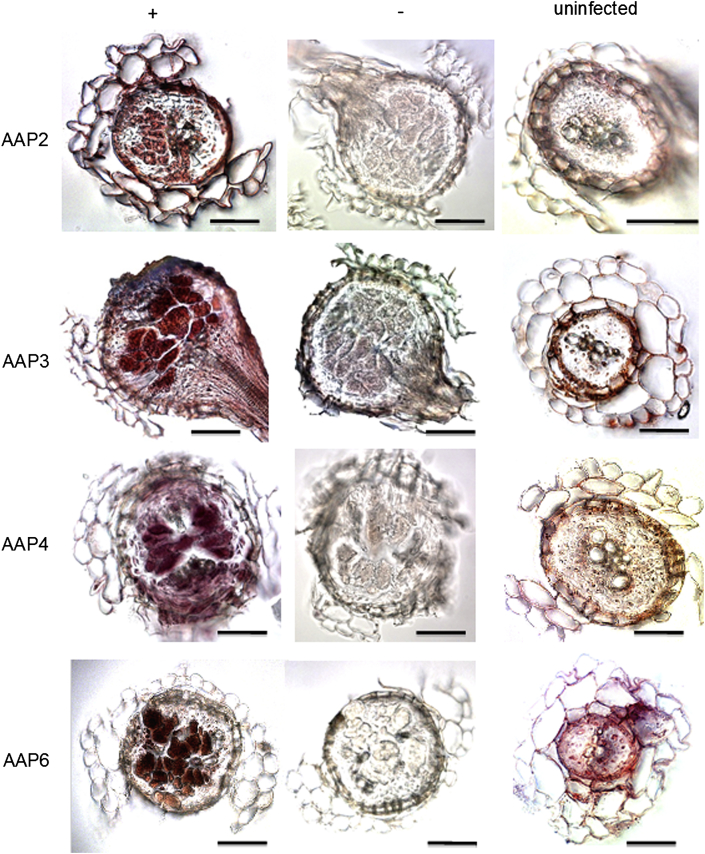
*In situ* RT-PCR for *AAP* genes. (+) = 15 dpi syncytia, (−) = negative control of 15 dpi syncytia, and uninfected = uninfected roots. The gene name on the left of each row shows the targeted gene (scale bar = 50 μm).

We have recently reported the *in situ* RT-PCR detection of *AAP8* expression in syncytia [7]. Here we have used *in situ* RT-PCR to study the expression of *AAP2*, *AAP3*, *AAP4*, and *AAP6* (Fig. 2). High levels of *AAP2*, *AAP3*, *AAP4*, and *AAP6* transcripts were detected mainly in syncytia and the central cylinder. In the cross-sections from uninfected roots, the expression of all tested *AAPs* was localized to the central cylinder with a different level of intensity. *AAP3* showed the highest level of expression in the central cylinder. Furthermore, *AAP3* expression was observed in the endodermis and the cortex. No specific staining was detected in the negative controls.

### 2.3 Resistance tests

**Fig. 3:**
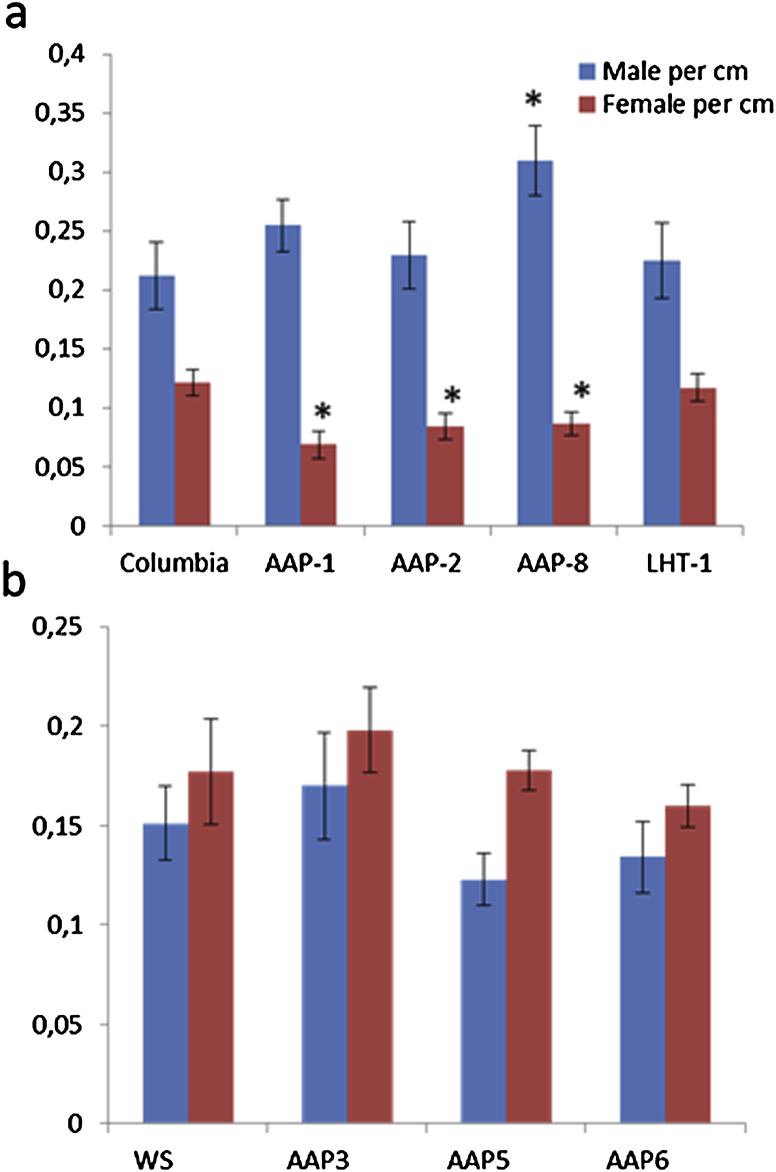
Nematode infection assay for *aap* and *lht1* mutants. Average numbers of females and males per cm root are shown with the standard error bar. The data are the average of all three independent repetitions. (a) Shows the results of the mutants with a Col genetic background and (b) shows the mutants with Ws genetic background. (*) marks the values with statistically significant difference to the wild type according to the LSD test at confidence level 90.0%.

The strong expression of the amino acid transporter genes that we have studied pointed to an important function of these genes for syncytium function and development. We have therefore tested the effect of knock-out mutants for the development of *H. schachtii*. The available mutants were in two different backgrounds (Table S1). The tests were therefore performed in two different groups with either the Col-0 or the Ws wild type. The first group included the *aap1*, *aap2*, *aap8*, and *lht1* mutants which were compared to the Col-0 ecotype as a control (Fig. 3a). The second group included the *aap3*, *aap5*, and *aap6* mutants and these were compared to the Ws ecotype (Fig. 3b). The mutant lines *aap1*, *aap2*, and *aap8* from the first group showed a significant difference in the number of females per cm root length but no significant difference was observed for the *lht1* mutant. The number of males was not significantly different for all mutants of the Col-0 background except in case of *aap8*. The *aap3*, *aap5*, and *aap6* mutants of the Ws wild type did not have a significantly different number of females as compared to Ws. The number of males on the *aap3*, *aap5*, and *aap6* mutants was also not significantly different from the wild type.

## 3 Discussion

### 3.1 Expression of amino acid transporter genes in syncytia

We have studied here the expression of several *AAP* genes and of *LHT1* in syncytia induced by *H. schachtii* in Arabidopsis roots using promoter::GUS lines and *in situ* RT-PCR. In general, the data reported here support the results of the transcriptome analysis of syncytia [7] with a few exceptions. For the *AAP1*::GUS line we did not find GUS expression in syncytia and also the expression in uninfected roots was restricted to the primordia of side roots and root tips. According to the syncytium transcriptome analysis this gene was upregulated in syncytia as compared to control root sections [7]. According to Genevestigator, a repository of GeneChip data [35] *AAP1* showed a very low expression level in roots (Fig. S4) while a root transcriptome analysis [36] found quite strong expression in the root central cylinder (Fig. S5). The reason for this discrepancy is currently unknown, but since the expression in uninfected roots and in syncytia was lower than could have been predicted from some transcriptome data, it might be possible that the promoter fragment that was used for the construction of the *AAP1*::GUS line, was too short. Unfortunately, the expression in roots of the promoter *AAP1*::GUS line was not reported in the original publication [22]. To resolve the discrepancies between these different transcriptome studies and the GUS analysis would require to produce GUS fusions with promoter fragments of different lengths.

In case of *AAP2* and *AAP4*, GUS expression was downregulated in syncytia at 15 dpi. The reason for this is currently unknown but might also be related to the promoter fragment that was used. Our *in situ* RT-PCR analysis has confirmed the presence of the *AAP2*, *AAP3*, *AAP4*, and *AAP6* transcripts in 15 dpi syncytia. In uninfected roots, the expression was mainly found in the central cylinder which is supported by data from the AREX database (Fig. S5).

### 3.2 Function of *AAP* transporter genes for cyst nematode development

The upregulation of most *AAP* genes in syncytia pointed to an important function of these genes for *H. schachtii* development. The majority of the 8 AAP amino acid transporters have moderate affinity for neutral and acidic amino acids while AAP3 and AAP5 also transport basic amino acids [9,11,37,38] and LHT1 was described as a lysine and histidine transporter [16]. Together, these transporters should be able to support the syncytium with all amino acids.

We tested several T-DNA mutants for their effect on nematode development. For 3 genes (*AAP1*, *AAP2*, *AAP8*) we found that a significantly lower number of females developed on the corresponding mutants. In case of *LHT1* and several other *AAP* genes (*AAP3*, *AAP5*, *AAP6*) no enhanced resistance was found although *LHT1* was strongly expressed in roots and syncytia and *AAP6* was the one with the strongest upregulation of all *AAP* genes. The reason that we did not find enhanced resistance in more single mutants is probably the redundancy among the large number of amino acid transporters. In the future it would therefore be necessary to combine several of the single mutants in one line to overcome this redundancy. The amino acid content of syncytia has been compared to uninfected root sections and it was found that the levels of several amino acids were higher in syncytia [32]. If the knock-out of certain amino acid transporters has an effect on the amino acid content of syncytia is not known. This would require a metabolic analysis of all mutants.

The mutants that we tested were in different backgrounds, Col and Ws. Sijmons et al. [39] tested 74 Arabidopsis ecotypes, including Col and Ws, and found that, for instance, Sah-0 and Lan-0 supported less females than Gre-0 and La-0. They did not publish the number of females per plant for Col and Ws, indicating that they were somewhere in between. In our work, Ws supported around 50% more females than Col. One might therefore argue that mutations in the Col background showed an effect because the Col plants are already less supportive for nematode development than the Ws ecotype because the *AAP* mutants in the Col background were more susceptible while those in Ws background were not. However, the LHT1 mutant which we tested was also in the Col background and this mutant showed no effect. Furthermore, the *AAP5* gene is downregulated in syncytia and it is therefore not surprising that the mutant for this gene, which is in the Ws background, did not show an effect. This indicates that the observed differences for the *AAP* genes are not just an ecotype effect. Unfortunately, nothing is known yet about the expression of *AAP* genes in the Ws ecotype. To rule out an ecotype specific effect, it would be necessary to analyse the same mutants in both ecotypes as has been done by Marella et al. [40] who tested *aap3* mutants in both ecotypes and found in both cases an effect on *M. incognita* development. The *aap3* mutant in the Ws background was the same mutant that we used but we did not see an effect on the number of *H. schachtii* males or females. Thus, AAP3 might be more important for gall nematodes than for cyst nematodes.

While the feeding site of cyst nematodes is the syncytium, feeding sites of gall nematodes contain several giant cells. In an analysis of transporter gene expression in Arabidopsis roots infected with *M*. *incognita*, only *AAP6*, *AAP7*, and to some degree *AAP3* were found to be upregulated [33]. However, in that study whole root samples were used and the specific expression in giant cells might have been overlooked. Another amino acid transporter that was upregulated in root knots was AtCAT6 [34]. Mutants for this gene did not show a difference in resistance to *M. incognita*. In a more comprehensive analysis of gene expression in root knots in Arabidopsis [41], galls were cut out from the roots and analysed using the CATMA microarrays (*AAP4* not included). This analysis, different from the situation described here, showed only a slight upregulation of *AAP1*, *AAP2*, and *AAP6* in galls compared to control root sections. If this shows the real situation in giant cells is doubtful since the samples contained probably more gall tissues than giant cells. In addition to these publications, gene expression has also been studied in microdissected giant cells [42]. The authors of that study classified *AAP6* as “gall distinctive”, meaning that it was “upregulated in galls but not in giant cells”, while *AAP5* was classified as “downregulated in galls and giant cells” as compared to vascular cylinder cells. Other *AAP* genes and *LHT1* were not analysed in that work.

Mutants for AAP3 and AAP6 transporters were analysed by Marella et al. [40]. It was found that both mutants had a significant effect on *M*. *incognita* development. Egg masses produced on the mutant roots were lower than on wild type roots, however, the double mutant did not differ from the single mutants, which lead the authors to speculate that both transporters might act in a coordinated fashion.

## 4 Conclusion

While sugar transport into syncytia has been intensively studied, not much is known about amino acid transport into syncytia. Our analysis has shown that AAP transporters play an important role in that regard, considering the strong expression in syncytia and the fact that some single mutants had a significant effect on nematode development. Since the specificity of AAPs for amino acids is to some degree redundant, it will be interesting to extend this analysis to combinations of single mutants and to combine this with a metabolic analysis.

## 5 Methods

### 5.1 Plant cultivation

Arabidopsis plants (mutants and Col-0 and Ws ecotypes) were cultured on 0.2% Knop medium containing 2% sucrose [39] for nematode infection experiments. Seeds were surface sterilized by soaking them in 5% (W:V) sodium hypochlorite for 10 min followed by 3 washes with sterile water. The plates were sealed and incubated in a growth chamber at 25 °C with 16 h light and 8 h dark cycles. The mutants that were used in this work are listed in Table S1.

### 5.2 Statistical analysis of microarray data

Affymetrix CEL files were analysed using packages of the Bioconductor suite (www.bioconductor.org). Details are provided in Ref. [7] and in the accompanying Online Supplement of that paper (Appendix S1). The additional online material for Szakasits et al. [7] providing large comprehensive tables and plots and detailed technical analysis results is archived at http://bioinf.boku.ac.at/pub/Szakasits2008/. A brief description is also provided in the Supporting Information (Appendix S1) of this paper.

### 5.3 Cloning of *pAAP4*::GUS

The *AAP4* promoter region was amplified by PCR using primers 5′- GAGATTGAGATGGGACCTCTGCG -3′ and 5′- GCTGGCCGTGGAACATCCATCTG -3′. The PCR fragment was cloned into the HincII site of pBluescript SK+, and was sequenced to ensure that no mutation had been introduced. The promoter sequence was then excised from the pBluescript SK+ vector with Bsp120I (subsequently blunted with Klenow enzyme) and XbaI, and cloned between the HindIII (blunted) and XbaI sites, of pGPTV-BAR [43] (upstream of the *uidA* sequence). The final plasmid was transformed into Agrobacterium GV3101 and introduced into Arabidopsis using *in planta* transformation [44]. Transformed Arabidopsis plants were selected on soil by spraying with BASTA. A single representative homozygous line was selected and used in this work.

### 5.4 *In situ* RT-PCR

*In situ* RT-PCR was carried out as described by Szakasits et al. [7]. Syncytia at 15 dpi were dissected from the roots and immediately immersed in cold fixation solution (63% ethanol, v/v; 2% formalin, v/v). After 24 h, syncytia were embedded in 4% low-melting agarose and 25 μm thick sections were prepared using a vibratome (VT 100, Leica, http://www.leica.com/). RT-PCR was then carried out using digoxigenin-labelled dUTP at the annealing temperature listed in Table S2 together with the primer sequences. After a staining reaction with nitro blue tetrazolium/5-bromo-4-chloro-3-indolyl phosphate substrate, cross-sections were photographed using an inverted microscope (Axiovert 200M, Zeiss, http://www.zeiss.com/) with an integrated camera (AxioCam MRc5, Zeiss, http://www.zeiss.com/).

### 5.5 Nematode infection assays

Cysts of *H. schachtii* were harvested from sterile *in vitro* stock cultures that were grown on mustard (*Sinapis alba* cv. Albatros). The collected cysts were soaked in 3% ZnCl_2_ at 25 °C for 4 days in order to hatch them. The collected juveniles were used for inoculating the 12 days old Arabidopsis seedlings (60 J2/plants) that were growing on 0.2% Knop medium (10 seedlings/plate). Root length was estimated at the date of inoculation as described by Jürgensen [45]. Each infection assay had 5 replicates from each T-DNA insertion line and the corresponding wild type (Col-0 for *aap1*, *aap2*, *aap4*, *aap5*, and *lht1*, and Ws for *aap3*, *aap5*, and *aap6*, respectively). Each mutant was at least tested in 3 independent experiments. Two weeks after inoculation, the number of males and females was counted in each plate. The pathogenicity level was calculated based on the number of male and female nematodes per cm of root length. For the statistical analysis we used STATGRAPHICS Centrion XV (Version 15.2.11) to perform a one-way ANOVA for the numbers of males and females per cm root length among mutants and wild types corresponding to their genetic background (Col or Ws). Box and whisker plot was used to detect outliers and identified outliers were removed from the analysis. The distribution of tested values of male- and female-infection was detected to make sure that the tested values are normally distributed (alpha = 0.1). Fisher's least significant difference (LSD) procedure was used to discriminate among the means defining the significantly different means (confidence level = 90,0%).

### 5.6 GUS assays

Transgenic Arabidopsis lines containing promoter::GUS fusions were grown as described above. All lines were infected with nematodes as described before and stained for GUS activity at 5 and 15 dpi. The samples were soaked in pre-chilled 90% acetone for 4 h followed by several washes with chilled distilled water. The specimens were then stained by overnight incubation at 37 °C in 100 mmol NaPO_4_ buffer (pH 7.0) containing 10 mmol EDTA, 0.01% Triton X-100, 0.5 mmol K_3_(Fe(CN)_6_), 0.5 mmol K_4_(Fe(CN)_6_) and 1 mg ml^−1^ 5-bromo-4-chloro-3-indolyl glucuronide. At the 5 dpi stage the nematodes were also stained by incubation in Fuchsin solution (875 ml lactic acid, 63 ml glycerol, 62 ml H_2_O, 0.1 g acid fuchsin) overnight at room temperature. Fuchsin staining made it easier to observe the juveniles at 5 dpi. At last, samples were washed several times with 70% EtOH followed by a final wash with 95% EtOH. The GUS lines that were used in this work are listed in Table S1.

## Figures and Tables

**Table 1:** Expression of genes coding for amino acid transporters in syncytia and control root sections according to microarray data. The data for microaspirated syncytia at 5 dpi and 15 dpi were compared with control roots (elongation zone without root tip was used as control). The third and fourth columns show the normalized expression values on a log_2_ scale. The differences (fold changes) between the pairwise samples displayed (fifth column) are accordingly normalized log_2_ ratios (see “Methods” section and the “Supporting methods” section in the Online supplement for details). The *q*-values in column 6 indicate significance after correction for multiple testing controlling the false discovery rate. Raw data are from Ref. [7].

Gene	ID	Control	Syncytium (5 + 15 dpi)	Syncytium *vs* control	*q*-value
*At1g58360*	AtAAP1	5.7	8.4	2.7	0.05
*At5g09220*	AtAAP2	9.9	11	1.1	0.32
*At1g77380*	AtAAP3	7.1	9.8	2.7^a^	0.00
*At5g63850*	AtAAP4	6.6	9.8	3.2	0.06
*At1g44100*	AtAAP5	7.8	5.2	−2.6^a^	0.00
*At5g49630*	AtAAP6	3.8	11.5	7.7^a^	9.26e−05
*At5g23810*	AtAAP7	5.2	4.1	−1.1^a^	0.02
*At1g10010*	AtAAP8	2.5	7	4.5^a^	3.41e−05
*At2g38120*	AtAUX1	6.2	6	−0.2	0.50
*At5g01240*	AtLax1	5.4	4.7	−0.7^a^	0.02
*At2g21050*	AtLax2	3	2.5	−0.5	0.04
*At5g40780*	AtLHT1	11.2	9.4	−1.8^a^	0.00
*At1g24400*	AtLHT2	2.6	2.5	−0.1	0.52
*At1g61270*	AtLHT3	not available on GeneChip			
*At1g47670*	AtLHT4	5.9	5	−0.9^a^	0.00
*At1g67640*	AtLHT5	3.6	3.2	−0.4	0.17
*At3g01760*	AtLHT6	not available on GeneChip			
*At4g35180*	AtLHT7	3.4	3.6	0.2	0.42
*At1g71680*	AtLHT8	3.9	3.4	−0.5	0.09
*At2g39890*	AtProT1	6.8	7.4	0.6	0.20
*At3g55740*	AtProT2	4.4	4.6	0.2	0.45
*At2g36590*	AtProT3	2.9	2.7	−0.2	0.34
*At1g08230*	AtProT4	3.1	3.3	0.2	0.36
*At5g41800*	AtProT5	5.7	6	0.3	0.14
*At3g11900*	ANT1	6.7	6	−0.7	0.06
*At5g65990*	ANT2	4.5	6.9	2.4^a^	0.00
*At4g38250*	ANT3	8.6	7	−1.6^a^	0.00
*At2g41190*	AVT1L1	5.2	6.2	1	0.05
*At3g09340*	AVT1L2	not available on GeneChip			
*At3g09330*	AVT1L3	not available on GeneChip			
*At5g02170*	AVT1L4	3.3	3.1	−0.2	0.45
*At5g02180*	AVT1L5	3.1	2.8	−0.3	0.17
*At3g54830*	AVT1L6	not available on GeneChip			
*At2g39130*	AVT1L7	3.6	3.3	−0.3	0.22
*At5g15240*	AVT1L8	3.6	3.3	−0.3	0.20
*At3g28960*	AVT1L9	2.6	2.2	−0.4	0.04
*At1g80510*	SN1L1	4.1	3.8	−0.3	0.20
*At5g38820*	SN1L2	7.9	4.9	−3^a^	0.00
*At3g30390*	SN1L3	10.3	5.9	−4.4^a^	0.00
*At3g56200*	SN1L4	6	5.8	−0.2	0.6
*At2g40420*	SN1L5	5.2	4.6	−0.6	0.13
*At4g21120*	AtCAT1	4.4	4.6	0.2	0.43
*At1g58032*	AtCAT2	not available on GeneChip			
*At5g36940*	AtCAT3	7.8	6	−1.8^a^	0.00
*At3g03720*	AtCAT4	6.7	5.4	−1.3	0.03
*At2g34960*	AtCAT5	3.5	3.1	−0.4	0.24
*At5g04770*	AtCAT6	5.3	5.9	0.6	0.03
*At3g10600*	AtCAT7	3.3	3	−0.3	0.20
*At1g17120*	AtCAT8	6.5	5.6	−0.9^a^	0.00
*At1g05940*	AtCAT9	8.1	7	−1.1^a^	0.00
*At5g05630*	AtLAT1	3.5	3.7	0.2	0.43
*At3g13620*	ATLAT2	5.1	7	1.9^a^	0.00
*At1g31820*	AtLAT3	4.6	3.9	−0.7	0.07
*At1g31830*	AtLAT4	8.1	7.5	−0.6	0.22
*At3g19553*	AtLAT5	6.2	5.2	−1^a^	0.09
*At5g44370*	AtBNP1hom1	5.2	5	−0.2	0.38
*At2g29650*	AtBNP1hom2	3.6	6.3	2.7^a^	0.00
*At3g46980*	AtBNP1hom3	4.3	4.1	−0.2	0.52
*At2g38060*	AtBNP1hom4	4.8	4.6	−0.2	0.45
*At4g00370*	AtBNP1hom5	5.4	7.6	2.2^a^	0.00

a Indicates significant up- or downregulation (false discovery rate <5%).

## Footnotes

Appendix A Supplementary data related to this article can be found at http://dx.doi.org/10.1016/j.plaphy.2013.05.016.
