# Supplementary material for: The AAP gene family for amino acid permeases contributes to development of the cyst nematode Heterodera schachtii in roots of Arabidopsis
Source: Plant Physiol Biochem. 2013 Sep;70:379–86. doi: 10.1016/j.plaphy.2013.05.016 (PMC3737465; doi:10.1016/j.plaphy.2013.05.016)
Supplement: Supplementary file 1 [file mmc1.pdf]

SUPPORTING INFORMATION

Fig. S1 MA plot (syncytium vs. root) for amino acid transporter genes.

Fig. S2 MA plot (15 dpi syncytium vs.

5 dpi syncytium) for amino acid transporter genes.

Fig. S3 GUS staining of leaves of *AAP4* promoter::GUS line.

Fig. S4 Relative expression of *AAP* and *LHT1* genes according to Genevestigator (www.genevestigator.ethz.ch).

Fig. S5 Expression of *AAP* transporter genes in roots according to the AREX database.

Table S1 Transgenic Arabidopsis lines used in this study.

Table S2 Primers used for *in situ* RT-PCR.

Appendix S1 Supporting methods microarray analysis.

Fig. S1

MA plot (syncytium vs. root) for amino acid transporter genes.

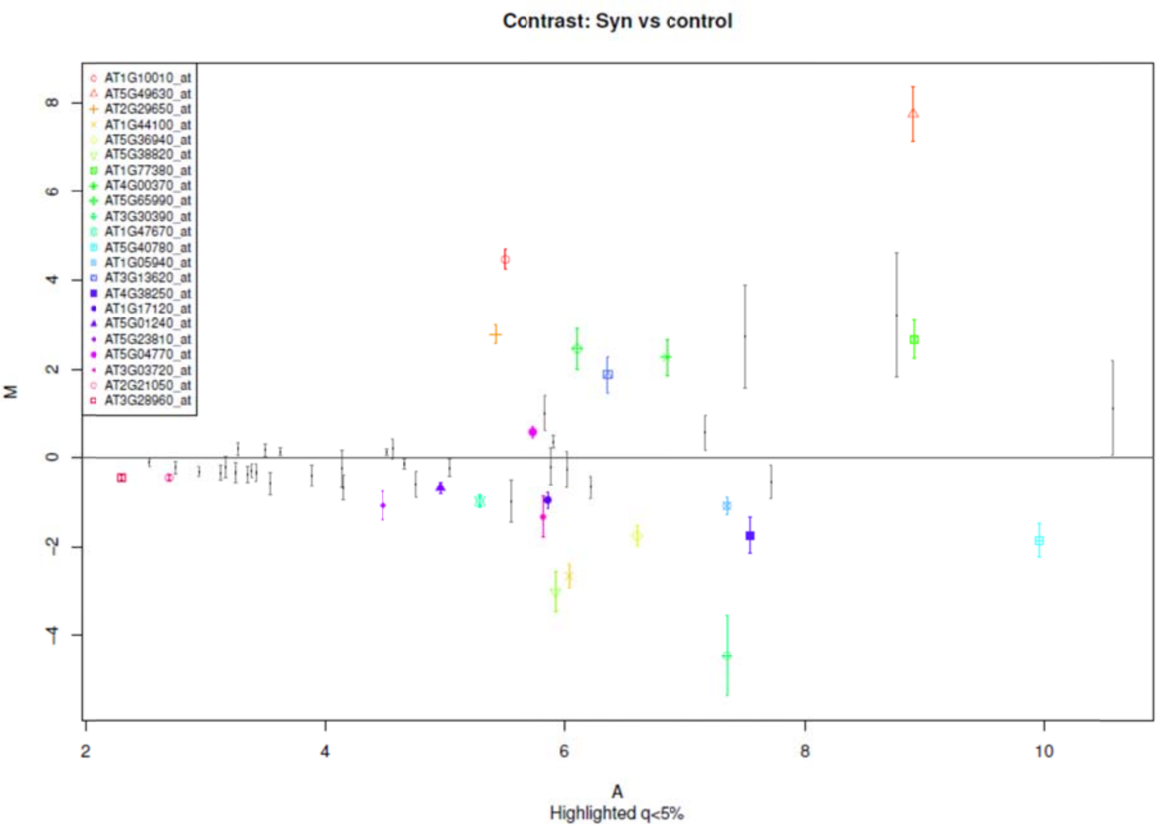

Fig. S2

MA plot (15 dpi syncytium vs. 5 dpi syncytium) for amino acid transporter genes.

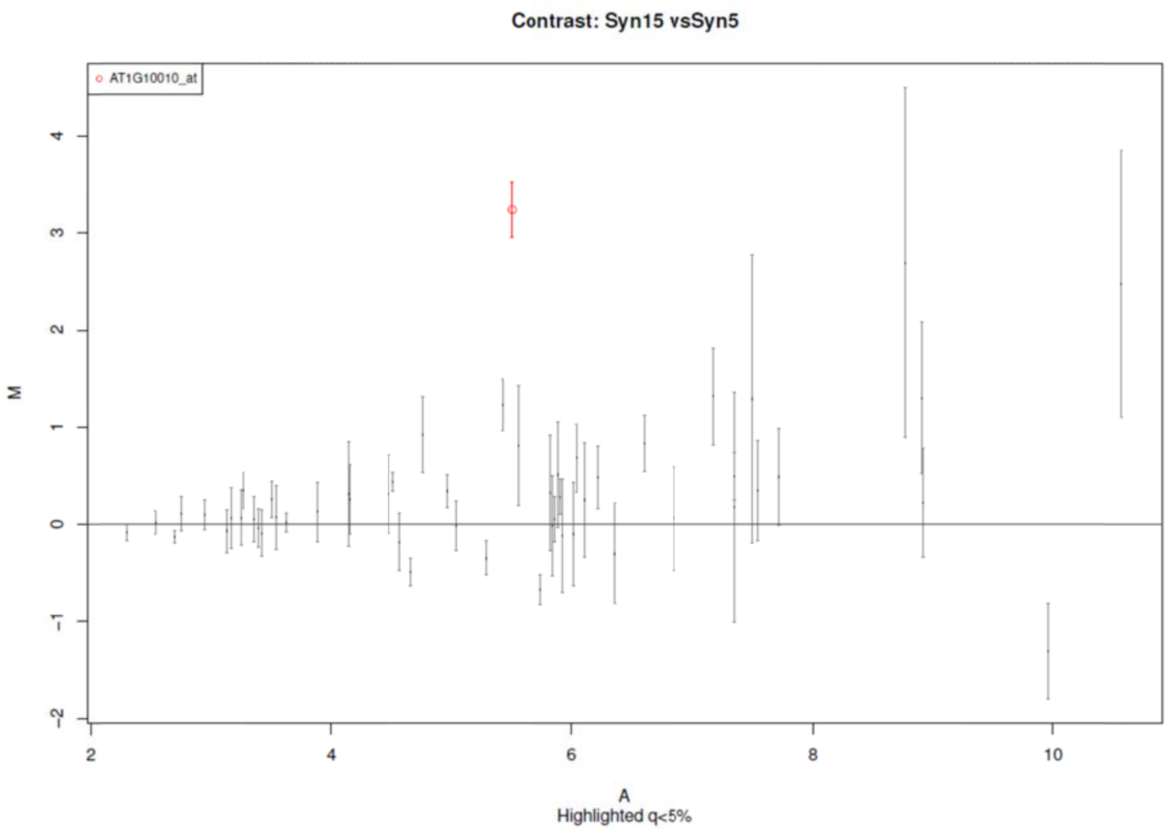

Fig. S3

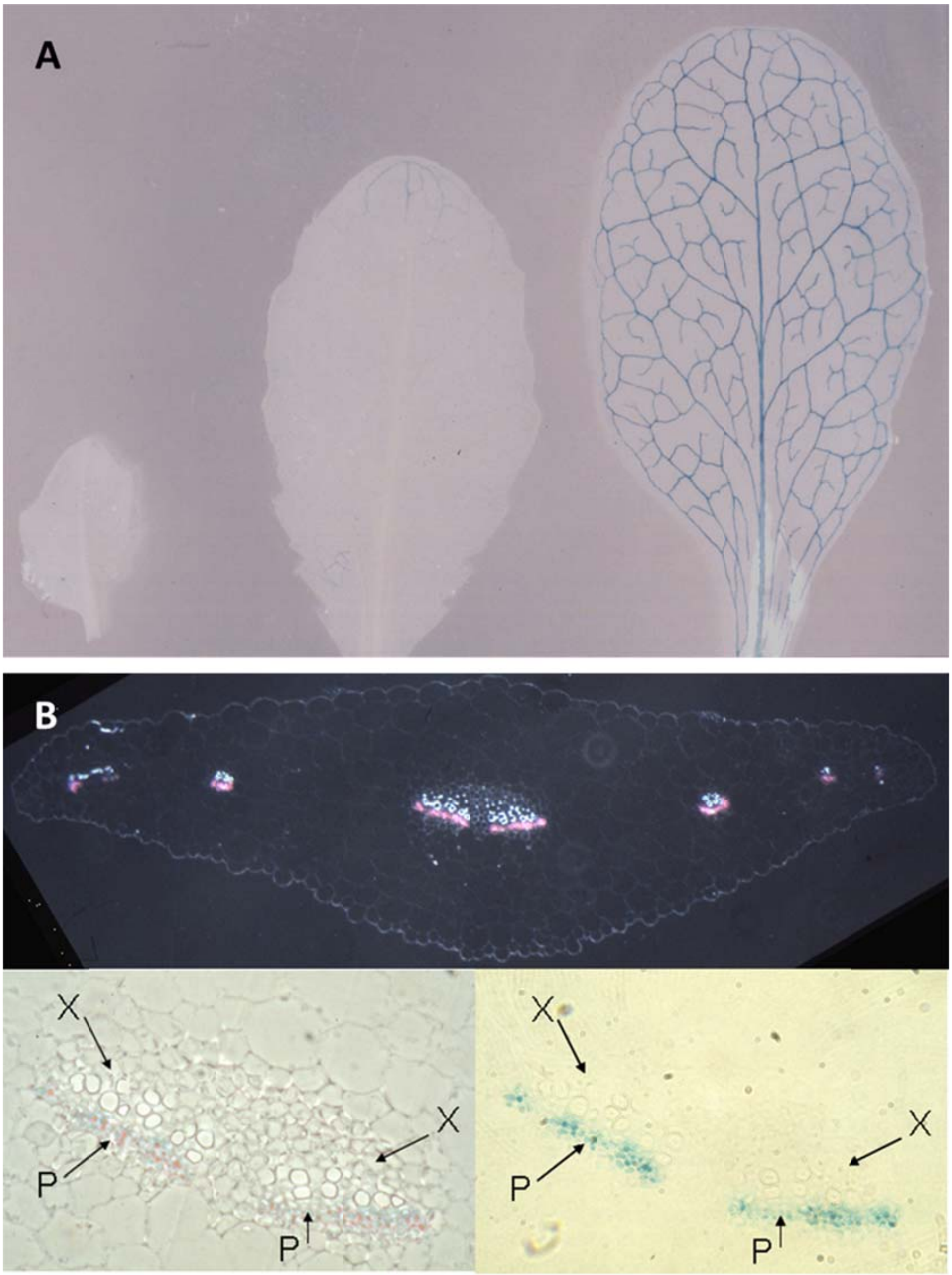

GUS staining of leaves of *AAP4* promoter::GUS line. GUS staining shows expression in the veins of older leaves (A). Cross sections (B) show that the expression is restricted to phloem cells. P, phloem; X, xylem.

Fig. S4

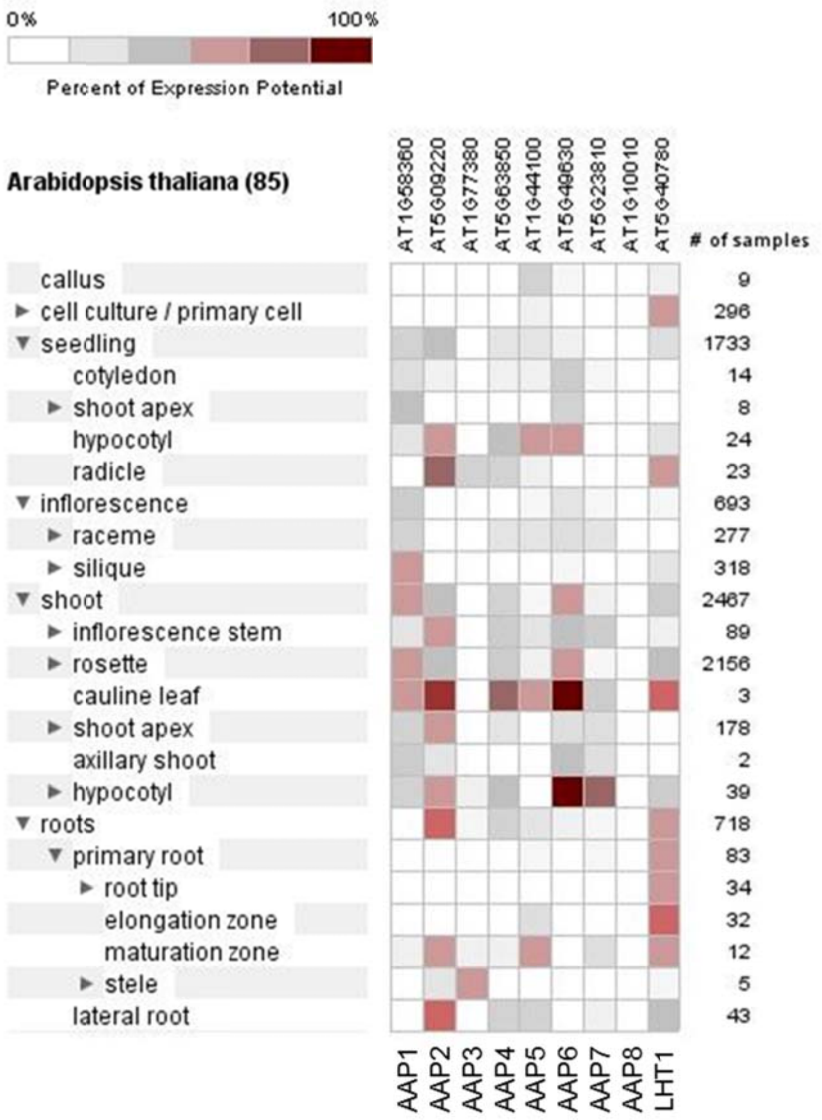

Relative expression of *AAP* and *LHT1* genes according to Genevestigator

(www.genevestigator.ethz.ch).

Fig. S5

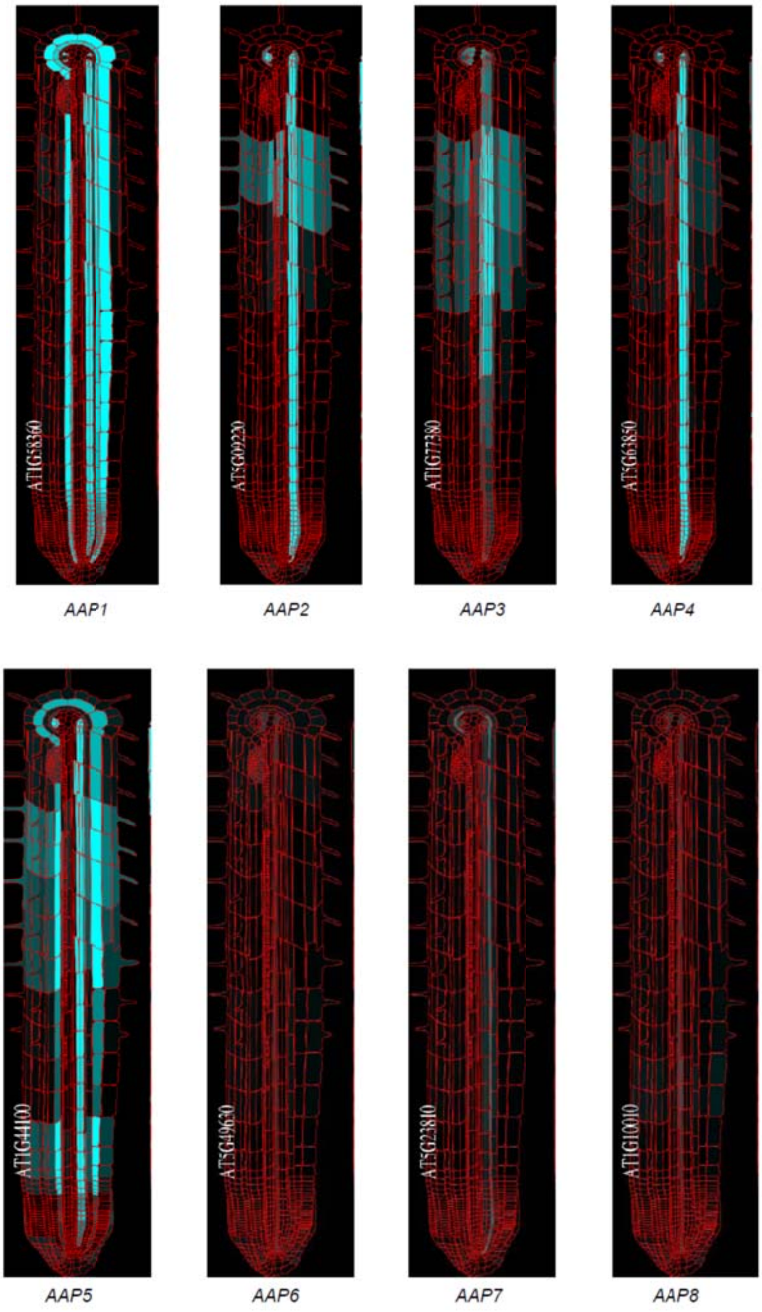

Expression of *AAP* genes in roots according to AREX database. Digital gene expression data for *AAP* genes from root transcriptome analysis (http://www.arexdb.org/index.jsp) using Affymetrix Arabidopsis GeneChips. Cyan colour indicates expression from low (light blue) to high (intense blue).

Table S1

Transgenic Arabidopsis lines used in this study

Gene	Gus line	KO mutant	KO background
AAP1	Hirner et al. 1998	Lee et al. 2007	Col
AAP2	Hirner et al. 1998	Zhang et al. 2010	Col
AAP3	Okumoto et al. 2004	Okumoto et al. 2004	Ws
AAP4	This work	-	
AAP5	-	Svennerstam et al. 2008	Ws
AAP6	Okumoto et al. 2002	Hunt et al. 2009	Ws
AAP7	-	-	
AAP8	Okumoto et al. 2002	Schmidt et al. 2007	Col
LHT1	Hirner et al. 2006	Hirner et al. 2006	Col

Table S2

Primers used for *in situ* RT-PCR.

Primer name	Sequence	Annealing temperature
**AAP2rev**	ACCTTAAGATCAAGCATCACTCC	54
**AAP2-IB-F**	GTTCAAGTTGCAGCGAATGGAGTT	54
**AAP3rev**	GTATTCGCTTCGAAATGGCTTGTAGG	55
**AAP3-IB-F**	CATCGAGATTCAGGACACAGTGAAG	55
**AAP4rev**	GAACGGCTTGTAAACCTTAAGGTC	53
**AAP4-IB-F**	GCATCTATAAGTATGATGGCGATCAAG	53
**AAP6for**	CAACACTGACAGGAGTTACGGT	55
**AAP6rev**	TTCGCGCTCTGGCTCTCTA	55

Appendix S1

Supplementary methods microarrray analysis

For the analysis of amino acid transporter gene expression in syncytia we used the eleven GeneChips described in Szakasits et al. (2009). Briefly, hybridizations were conducted by the German Resource Centre for Genome Research GmbH (now ATLAS Biolabs GmbH; both in Berlin, Germany) following the manufacturer's protocols (for details see Szakasits et al., 2009). Affymetrix CEL files were read into the R statistical analysis environment (www.r-project.org) using the *affy* package of the Bioconductor suite (www.bioconductor.org). As 10–40% of probe sets are affected by updated gene annotation, chips were processed with current TAIR v8 probe-set annotation (Dai et al., 2005). Probe sequence specific 'background correction' (Wu et al., 2004) was performed using routines available in the Bioconductor *gcrma* package. Using the 'affinity' model, while 'MM' probes were employed for the determination of affinity parameters, only 'PM' probes were used for the probe-specific background correction. An inspection of exploratory pairwise scatter and 'MA' plots confirmed the need for inter-chip normalization. The thus required explicit normalization steps made a subtraction of the heuristic estimate for optical instrument background as offered in gcrma unnecessary. Defaults were used for all other *gcrma* parameters. As an examination of pairwise quantile-quantile plots showed only random fluctuations, inter-chip normalization could be achieved using quantile-quantile normalization (Bolstad et al., 2003). See 'Low-level microarray analysis and diagnostic plots' section of the Online Supplement for diagnostic plots and Figures (Szakasits et al., 2009). After normalization, robust summaries of probe set signals were obtained for each gene using an iterative weighted least squares fit of a linear probe level model (Bolstad, 2004) through the *fitPLM* function of the Bioconductor package *affyPLM*. This process automatically identifies unreliable chip

areas and correspondingly downweights outlier probes. See Online Supplement of Szakasits et al., 2009 for Figures. The normalized data on log2 scale were then fitted gene by gene with a linear model including hybridization batch effects, using the *lmFit* function (Smyth, 2004) of the Bioconductor package *limma*. The result-tables also include *q*-values as indicators of significance of contrasts after correction for multiple testing controlling the False Discovery Rate (Benjamini and Hochberg, 1995).

For the statistical tests, individual gene variances have been moderated using an Empirical Bayes approach that draws strength from transferring variance characteristics from the set of all genes to the test for each individual gene (Smyth, 2004). Tests were restricted to the subset of 52 amino acid transporter genes that are included on the GeneChip. This considerably increases the statistical power of the testing procedure as it reduces the necessary correction for massive multiple testing.

Supplementary References

Benjamini, Y., and Hochberg, Y. (1995) Controlling the false discoveryrate: a practical and powerful approach to multiple testing. J. R. Stat. Soc. Ser. B **57**, 289–300.

Dai, M., Wang, P., Boyd, A.D., Kostov, G., Athey, B., Jones, E.G., Bunney, W.E., Myers, R.M., Speed, T.P., Akil, H., Watson, S.J., and Meng, F. (2005) Evolving gene/transcript definitions significantly alter the interpretation of GeneChip data. Nucleic Acids Res. **33,** e175

Wu, Z., Irizarry, R.A., Gentleman, R., Martinez-Murillo, F. and Spencer, F. (2004) A model based background adjustment for oligonucleotide expression arrays. J. Am. Stat. Assoc. **99**, 909–917.

Bolstad, B.M., Irizarry, R.A., Astrand, M., and Speed, T.P. (2003) A comparison of normalization methods for high density oligonucleotide array data based on bias and variance. Bioinformatics **19**, 185–193.

Bolstad, B.M. (2004) Low Level Analysis of High-density Oligonucleotide Array Data: Background, Normalization and Summarization. PhD Dissertation. University of California, Berkeley.

Smyth, G.K. (2004) Linear models and empirical Bayes methods for assessing differential expression in microarray experiments. Stat. Appl. Gene. Mol. Biol. **3**, Article 3.
